# Validation of the Swedish Eating Assessment Tool, S-EAT-10, for patients with head and neck cancer

**DOI:** 10.1038/s41598-025-97170-5

**Published:** 2025-04-16

**Authors:** Linnéa Ekström, Lotta Sjökvist Wilk, Caterina Finizia, Lisa Tuomi

**Affiliations:** 1 Department of Health and Rehabilitation, Institute of Neuroscience and Physiology, Sahlgrenska Academy, University of Gothenburg, Gothenburg, Sweden; 2 Department of Otorhinolaryngology, Head and Neck Surgery, Institute of Clinical Sciences, Sahlgrenska Academy, University of Gothenburg, Gothenburg, Sweden; 3 Department of Otorhinolaryngology, Head and Neck Surgery, Region Västra Götaland, Sahlgrenska University Hospital, Gothenburg, Sweden

**Keywords:** EAT-10, Validation, Patient reported outcome measures, Dysphagia, Head and neck cancer, Oncology, Nutrition, Quality of life

## Abstract

The aim of this study was to validate the Swedish version of Eating Assessment Tool (S-EAT-10) for head and neck cancer patients. The participants (n = 60) had persistent swallowing difficulties 6–36 months after completion of curative radiotherapy. The videofluoroscopic swallowing study was assessed using the Penetration Aspiration Scale and the Yale Pharyngeal Residue Severity Rating Scale modified for videofluoroscopy. Participants completed questionnaires S-EAT-10, M.D. Anderson Dysphagia Inventory (MDADI) and study-specific questions. Internal consistency was excellent and the test–retest reliability was good. Regarding convergent validity, S-EAT-10 showed moderate to strong correlation with the MDADI and no to weak correlation with study-specific questions regarding meal duration and weight change. Regarding criterion validity, there was a weak correlation between S-EAT-10 and instrumental measures. S-EAT-10 showed 85% sensitivity in identifying patients with dysphagia. S-EAT-10 could not discriminate between different degrees of dysphagia. Thus, S-EAT-10 showed sufficient psychometric properties regarding head and neck cancer patients.

## Introduction

Swallowing difficulties, i.e. dysphagia is commonly occurring among elderly^1–4^ and as a consequence of illness, such as stroke, or head and neck cancer (HNC). As many as up to 40–70% of HNC patients experience some level of dysphagia following oncologic treatment^5–7^. Dysphagia encompasses reduced effectiveness and safety in swallowing, with an increased risk of malnutrition, dehydration, airway obstruction, aspiration pneumonia and weight loss^8^. Further, it is known that a greater weight loss may increase the risk for severe dysphagia in irradiated HNC patients^9^. Another consequence of swallowing difficulties may be prolonged meal duration, which further increases the risk for malnutrition^10^. Further, dysphagia has consequences not only in the physical aspect but also social and emotional impact, with worry of choking, embarrassment around eating in front of others, and frustration in being limited in their eating, which may result in isolation and avoidant behavior. All of those aspects affect the health-related quality of life (HRQL)^8^.

The number of cases of HNC are increasing worldwide and in Sweden, which is expected to lead to an increase in the number of radiotherapy patients and thus the number of patients with dysphagia^8,11^. Physical, functional, emotional and psychosocial aspects are affected in dysphagia, which in turn affect patient’s quality of life^8^. Several instruments concerning swallowing difficulties in different patient cohort exist, such as the Swallowing quality of life (Swal-QOL)^12^, Dysphagia Handicap Index^13^ and the M.D. Anderson Dysphagia Inventory (MDADI)^14,15^. Further, the Eating Assessment Tool (EAT-10) is used as a screening instrument in clinical practice around the world^16^ and is described as easy to administer and time-efficient for both patients and health professionals^17^. The EAT-10 has been validated in several languages and shows good psychometric properties^18–21^. The Swedish translation of the EAT-10 (S-EAT-10) has been validated in a mixed population of patients with dysphagia^18^. The consistency of the instrument with equivalent scales, i.e. convergent validity appears to be somewhat unexplored, and the S-EAT-10 has not yet been validated for HNC, still lacking information about criterion validity through comparison with instrumental measures of swallowing function^18^.

Therefore, the aim of the present study was to perform psychometric analyses of the Swedish translation of the EAT-10 through analysis of reliability and validity in a head and neck cancer population with dysphagia following radiotherapy.

## Method and material

### Participants

**Fig. 1:**
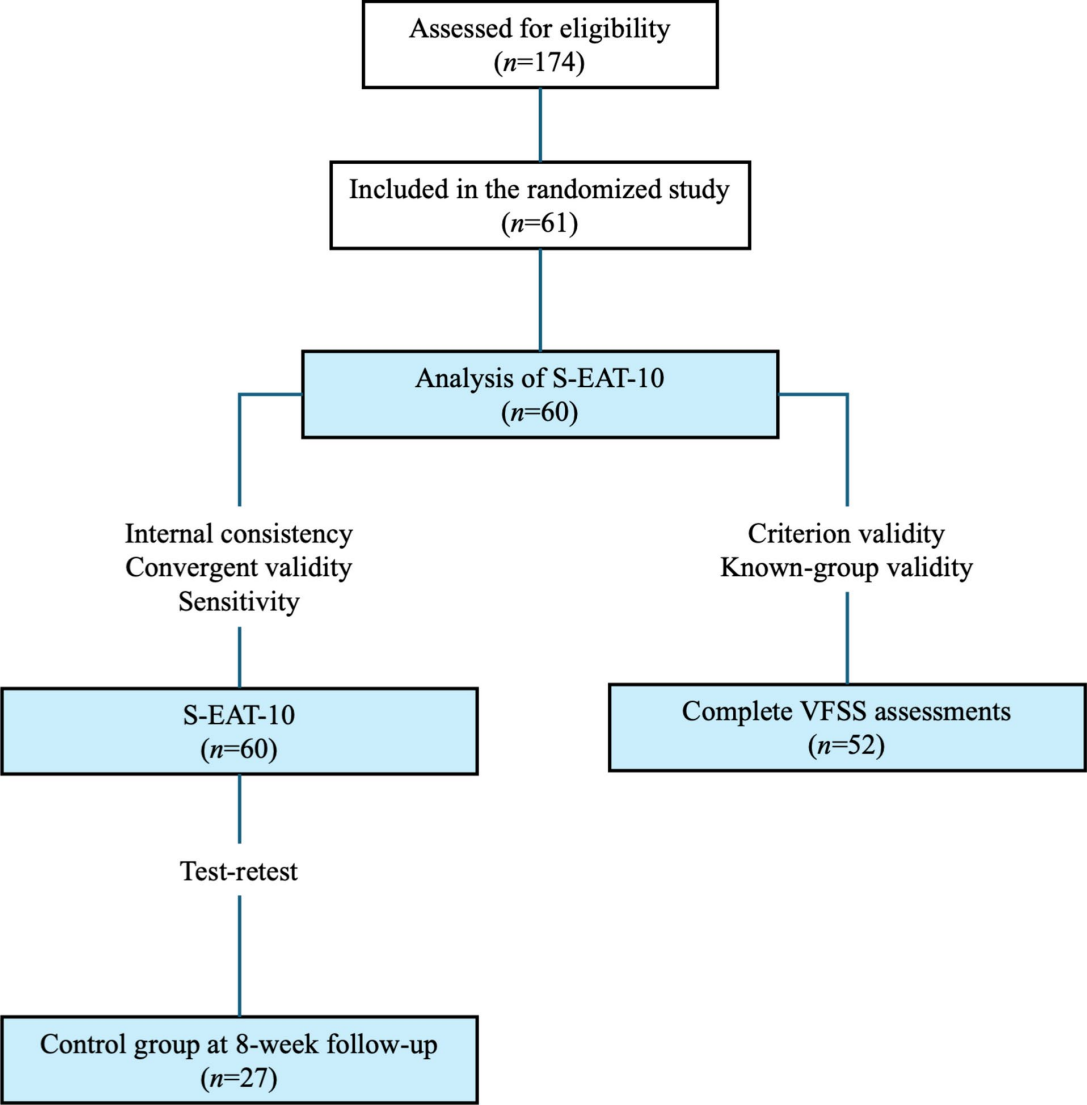
Flow chart over participants included in the respective analysis. Further information regarding included and excluded participants are found in the original publication of the randomized study.

All participants in this study were included in a randomized study evaluating the effectiveness of the head-lift exercise, which has been reported previously^22–24^. Patients diagnosed with HNC in Region Västra Götaland were discussed at the weekly multidisciplinary tumor board meeting at Sahlgrenska University Hospital (Gothenburg, Sweden). Patients > 18 years of age who were treated with curatively intended radiotherapy with or without chemotherapy for tumors of the tonsil, base of tongue, hypopharynx or larynx with no previous history of dysphagia were eligible for inclusion. Patients were asked to participate in a videofluoroscopic swallow study (VFSS) between 6 and 36 months following oncologic treatment, and those with remaining dysphagia (i.e. Penetration Aspiration score (PAS)^25^ score of ≥ 2) were eligible for inclusion in the randomized study. The time frame 6–36 months was selected to include patient where the side effects such as dysphagia are generally considered as late, aiming to not include patients with acute side effects from treatment. Inclusion of PAS ≥ 2 was selected in order to include patients with a wide range of difficulties. Patients were excluded if they had received surgery for HNC (except tonsillectomy or diagnostic sample excision), previous radiotherapy or other treatment for HNC, tracheostomy, neurological or neuromuscular disease, inability to swallow any bolus, and/or inability to perform the intervention. Patient inclusion and data regarding oncologic treatment at follow-up is reported elsewhere^22–24^, reporting on outcomes from a randomized controlled study, where the intervention group performed the HLE during 8 weeks, and the control group received dysphagia management in terms of texture modification and postural changes. Both intervention and control group were eligible for inclusion in the present analysis, if they had completed the S-EAT-10 at baseline, resulting in a total of 60 participants (Fig. 1). To allow for calculation of test–retest analysis the participants in the control group were compared at baseline and the 8-week follow-up, based on our previous reports that they did not reveal any significant changes of swallowing function (PAS)^23^ and were therefore included in the test–retest analysis (n = 27). The control group were similar to the intervention group in terms of tumor site and size, age, sex and comorbidity, as described elsewhere^23^.

Data was collected at baseline (before intervention) for all measures, where the VFSS and completion of study questionnaires were performed at the same time. The 8-week follow-up data for the S-EAT-10 was used for the control group allowing for test–retest calculation. A commonly used guideline concerning sample size estimation, is that it is necessary to have between 5 and 10 respondents for each item in the questionnaire^26^, resulting in the need of between 50 and 100 participants in the validation of the S-EAT-10.

### S-EAT-10

The EAT-10 consist of ten items regarding common symptoms and complaints among people with dysphagia. Each item is answered using a 5-point Likert scale from no difficulty (0) to severe difficulty (4). A total sore is calculated from the sum of all responses, and a score of 3 or more is considered abnormal^17^. The EAT-10 was translated into Swedish (S-EAT-10) by Möller et al.^18^ and is commonly used in different patient groups as a tool to evaluate the prevalence of dysphagia^16,27–29^. The instrument has been found valid in several languages^16^, including Swedish^18^.

### M.D. Anderson dysphagia inventory (MDADI)

The MDADI is a patient reported outcome measurement (PROM) which values how dysphagia impacts the health-related quality of life in patients treated for HNC^15^. It consists of 20 items divided into four domains and an overall score. The Global domain concern how the swallowing function limits everyday activities. The Emotional domain regard items of the patient’s affective response to their difficulties while the Functional domain highlights how the swallowing problem affect the patient’s private and social life. The physical domain concerns how the patient perceives their difficulty. Each item is rated on a Likert scale ranging from strongly agree (1) to strongly disagree (5). The domain scores are calculated by multiplying the mean of the domain scores by 20, and a total score including all items except the global question. Each item therefore ranges from 20 to 100 where the higher score represents a better HRQL. The MDADI has been translated into Swedish demonstrating retained psychometric properties^14^, and demonstrating the ability to capture changes over time^30^

#### Study specific items

Patients also responded to questions regarding meal duration in minutes and weight change, which was calculated as difference from the lowest weight during disease until VFSS, which was then transformed into percent weight change.

### Assessment of swallowing function during the VFSS

VFSS was performed in collaboration between a radiologist and an SLP. The methodology and boluses given are described in detail elsewhere^23^. All boluses given were analysed using the PAS and the Yale Pharyngeal Residue Severity Rating Scale modified for videofluoroscopy. The PAS^25^ is a commonly used and validated scale describing the extent and depth of aspiration and penetration, ranging from 1 (normal) to 8 (silent aspiration). The residue in the vallecula and pyriform sinus was assessed using a modified version of the Yale Pharyngeal Residue Scale for VFSS, with a rating scale from 1 (no residue) to 5 (more than 50% of the estimated volume of the vallecula and pyriform sinus)^31,32^. Each bolus was assessed separately, but only the overall worst scores were used in the present study.

### Statistical analysis

Statistical analyses were performed using the IBM SPSS (Statistical Package for the Social Sciences) version 29.0.2.0. Due to the assumption of data not being normally distributed, and several outcome measures being ordinal level data, mainly non-parametric tests were used in the analysis.

Internal consistency was calculated with the Cronbach’s alpha (α), where estimates > 0.7 were taken to indicate sufficient internal consistency reliability^26^. Test–retest reliability was assessed by intraclass correlation (ICC) and Spearman rank correlations (*rho*) for participants completing the S-EAT-10 twice. This was calculated for the control group who in our previous publications have been found to not change significantly regarding swallowing function during this period of time^23^. Values of ICC between 0.50 and 0.74 were considered acceptable, 0.75–0.90 good, > 0.90 excellent reliability^33^ while *rho* values of 0.25–0.50 were considered weak correlations, 0.50–0.75 moderate and > 0.75 strong correlation^34^.

Convergent and criterion validity was also calculated using the Spearman rank correlation (*rho*). Convergent validity was assessed by comparing the results from the S-EAT-10 with the MDADI domains and total score. Criterion validity was assessed by correlating the scores of S-EAT-10 with reference measures of objective evaluation of swallowing function from the VFSS. We hypothesized a priori that there would be at least moderate correlations between the S-EAT-10 and the MDADI domains, with the strongest correlation to the Physical domain, considering the similarity between the items of the domain and the items of the S-EAT-10. To compare groups with different degrees of dysphagia, the Mann Whitney U test was used comparing S-EAT-10 scores between the groups. The groups were divided by level of PAS, where PAS 2-4 indicated mild dysphagia and PAS 5-8 indicated moderate to severe dysphagia based on previous work in dysphagia in HNC^9^. Further, we expected weaker correlations to the objective measures of swallowing function, due to previous studies finding correlations between the EAT-10 and the PAS and Yale Pharyngeal Residue Scale^28,35^.

### Ethical considerations

The study was performed according to the Declaration of Helsinki and was approved by the Regional Ethical Review Board in Gothenburg, Sweden. All participants received oral and written information and provided their written informed consent prior to inclusion in the study. To ensure the protection and privacy of the participants, all data was de-identified and coded.

## Results

Participant characteristics are found in Table 1. Median age of the participants were 63 years, and the majority (73%) were men. Tumors of the tonsil were the most common localization (40%), and a majority were stage IV tumors (70%).

**Table 1:** Participant characteristics.

		Median (Min.–Max.)
Age (years)		63 (44–80)
Time since completion of oncologic treatment (months)		9 (6–37)
		n (%)
Sex	Male	44 (73)
	Female	16 (27)
Education	Elementary school	20 (33)
	High school	21(35)
	Higher education	18 (30)
	Missing	1 (2)
Self-perceived difficulties swallowing/eating/drinking or coughing during meals		50 (83)
Meal duration (min)	< 15 min	9 (15)
	15–30 min	39 (65)
	30–45 min	6 (10)
	> 45 min	2 (2)
	Not able to swallow	3 (5)
	Missing data	1 (2)
Feeding tube at time of VFSS		7 (12)
Comorbidity (ACE-27)^a^	None	32 (53)
	Mild	20 (33)
	Moderate	7 (12)
	Severe	1 (2)
Body mass index (BMI) classification^a^	Underweight (< 18.5)	2 (3)
	Normal (18.5–24.9)	42 (70)
	Overweight (> 25.0)	12 (20)
	Missing	4 (7)
Tumor localization	Tonsil	24 (40)
	Base of tongue	21 (35)
	Hypofarynx	8 (13)
	Larynx	7 (12)
Tumor stage	I	6 (10)
	II	6 (10)
	III	6 (10)
	IV	42 (70)

ACE-27 = Adult Comorbidity Evaluation-27^50^.

^a^Classification according to the World Health Organization.

The participants in the present study demonstrated a mean score of the S-EAT-10 at 14 (*SD* = 10; range 0–35). Results of the calculation for internal consistency demonstrated a Cronbach’s alpha of 0.92, which is considered excellent. The test–retest reliability showed good reliability with (ICC = 0.88) and a strong correlation between the first and second administration of the questionnaire (*rho* = 0.89, *p* < 0.001).

The comparison between S-EAT-10 scores and reference measures are found in Table 2. The correlations to the MDADI domains were generally strong, with the highest correlation being found in the Physical domain and total score. The correlations to objective measures from the VFSS showed statistically significant, however weak, correlations.

**Table 2:** Correlation coefficients between the total score of S-EAT-10 and reference measures of swallowing function.

	S-EAT-10
MDADI domains	
Emotional	− 0.81**
Functional	− 0.83**
Physical	− 0.88**
Global	− 0.73**
MDADI total	− 0.90**
Measures of VFSS	
Penetration aspiration scale	0.34*
Residue in vallecula	0.46**
Residue in pyriform sinuses	0.32*

*MDADI* M.D. Anderson Dysphagia Inventory. *VFSS* Videofluoroscopic evaluation of swallowing function.

*p < 0.05.

**p < 0.001.

The analysis of S-EAT-10 and meal duration showed a statistically significant weak correlation (*rho* = 0.36, *p* < 0.05). The analysis comparing S-EAT-10 scores and weight change did not show a statistically significant correlation *(rho* = − 0.24, *p* = 0.07).

In order to calculate the known-group validity, the participants were divided into groups of mild dysphagia (PAS 2-4) and moderate-severe dysphagia (PAS 5-8). The results showed no statistically significant difference between the groups regarding scores of S-EAT-10. The participants in the mild dysphagia group (n = 31) had a mean score of 24.7 scores while the moderate-severe dysphagia group had a mean score of 29.2.

Sensitivity was evaluated, considering all participants were considered as having dysphagia to some extent, and using the previously established threshold indicating dysphagia of ≥ 3 points, the analysis revealed an 85% sensitivity, i.e. 51 participants demonstrated a S-EAT-10 score of three or more.

## Discussion

The aim of the present study was to evaluate the psychometric properties of the S-EAT-10 for a Swedish HNC cohort. Results demonstrated sufficient reliability and validity. Internal consistency refers to how coherently different aspects or parts of an instrument measure the underlying theoretical concept^26^, in this case how well the questions in the S-EAT-10 correlate and thus measure dysphagia. Internal consistency was excellent in the present study which is in line with previous validations who demonstrated Cronbach’s α ranging between 0.84 and 0.96^17,18,27–29,35–38^ with α > 0.9 for HNC populations^39,40^. With this in mind, the EAT-10, and now also the S-EAT-10 is considered a reliable instrument, in terms of internal consistency, for a HNC population. The S-EAT-10 showed good test–retest reliability, i.e. the instrument’s ability to show the same results in a repeated measurement^34^. This result is in line with previous studies where ICC ranged between 0.84 and 0.99, and *rho* 0–92-0.99^17,18,28,35,37–39^.

Convergent validity concerns whether the instrument is consistent with equivalent scales and thus measures the theoretical underlying concept^26^. In the present study this was analyzed by comparing the equivalence of S-EAT-10 scores with MDADI domains. The results were similar to what was found in the French validation^38^. These results indicate that the S-EAT-10, despite having only half the number of statements, captures the aspects covered in the MDADI. Overall, the results of this study point to good convergent validity.

Criterion validity is usually analyzed comparing the association to objective findings or a gold standard measurement. In the present study, we found weak correlations between the S-EAT-10 and the instrumental measures used as gold standard measurements. These results are overall in line with previous studies evaluation the associations between the EAT-10 and instrumentally assessed swallowing function^28,35,41–43^ as well as similar to reports saying that self-reported swallowing function seldom correlate to more objective measures of swallowing function in HNC patients^44–46^. The weak correlations may reflect that the S-EAT-10 not only capture physical and functional aspects such as experience of aspiration or residue, but also emotional, psychological and social matters as the original instrument was intended to^17^. Therefore, the S-EAT-10 may be considered as having sufficient criterion validity.

When comparing groups of different dysphagia severity the S-EAT-10 could not discriminate between the groups. This was to be expected, since the EAT-10 was developed to capture the prevalence, not discriminate between degrees of difficulties^17^, even though some studies have found the EAT-10 to predict aspiration in some patients with neurological diseases^47–49^. This would indicate that there may be a need of a more specific questionnaire that might be able to differentiate between dysphagia severity, such as the MDADI^15^. Regarding known-group validity in terms of discriminating between people with and without dysphagia, the EAT-10 has demonstrated statistically significant differences in scores between non-dysphagia and dysphagia populations^17,27–29,38,39^. The Swedish translation study reported the numbers for the non-dysphagia control group with a mean value of 0.2 (SD 0.6, range 0–3) which is well below the values of the present study, this would indicate that the S-EAT-10 has a good known-group validity in a HNC population.

This study may be limited by the long period of time between test and retest, which was in median 9 weeks, compared to around 7–15 days as reported in previous validation studies. This time interval is often chosen since no changes are expected to occur during this short period of time. The longer interval between testing and retesting in our study could mean that changes have occurred, which could affect whether or not the results are reliable. However, as previous studies on swallowing function in this patient cohort have not shown any significant changes in swallowing function as measured by instrumental measures^22,23^ this was considered acceptable. Another limitation may be the definition of dysphagia as PAS of at least 2 (i.e. penetration to the larynx), resulting in the study including a wide range of difficulties, from very mild difficulties to more severe dysphagia. Additionally, a higher limit such as PAS 3 or higher, might have yielded a different result when calculating the known-group validity. Further, one limitation may be that the participants of the study did not go through a formal testing of cognitive function, which is an important aspect to consider when evaluating the use of patient reported outcome measures. Future studies are recommended to include some type of screening for cognitive function, as suggested previously^18^.

## Conclusion

To conclude, the S-EAT-10 demonstrated excellent values of reliability, and good validity. The S-EAT-10 is an easily administered questionnaire suitable to identify the prevalence of dysphagia in HNC patients following radiotherapy. However, in cases where the scope is to evaluate different degrees of dysphagia, it might be more suitable to use a more extensive instrument.

## Data Availability

The research data cannot be shared publicly due to ethical reasons. Any questions are directed to the corresponding author, Lisa Tuomi.
